# The effect of radiation therapy on the mechanical and morphological properties of the enamel and dentin of deciduous teeth—an *in vitro* study

**DOI:** 10.1186/1748-717X-9-30

**Published:** 2014-01-22

**Authors:** Talitha de Siqueira Mellara, Regina Guenka Palma-Dibb, Harley Francisco de Oliveira, Francisco Wanderley Garcia Paula-Silva, Paulo Nelson-Filho, Raquel Assed Bezerra da Silva, Léa Assed Bezerra da Silva, Alexandra Mussolino de Queiroz

**Affiliations:** 1 Department of Pediatric Clinics, School of Dentistry of Ribeirão Preto, University of São Paulo, Ribeirão, Preto, Brazil; 2 Department of Pediatric Clinics, Faculty of Dentistry of Ribeirão Preto, University of São Paulo, Ribeirão, Preto, Brazil; 3 Department of Restorative Dentistry, School of Dentistry of Ribeirão Preto, University of São Paulo, Ribeirão, Preto, Brazil; 4 Department of Medical Clinics, School of Medicine, University of São Paulo, Ribeirão, Preto, Brazil

**Keywords:** Radiotherapy, 60 cobalt, Radiation caries, Microhardness, Primary teeth, Morphology

## Abstract

**Purpose:**

To evaluate the effects of radiation therapy on deciduous teeth.

**Materials and methods:**

The enamel and dentin microhardness (n = 12) was evaluated at 3 depths, both before (control) and after each 10 Gy of irradiation and up to a dose of 60 Gy. The morphology was evaluated via scanning electron microscopy (SEM) (n = 8). The data were analyzed using a two-way analysis of variance (ANOVA) and Tukey’s test (α = 5%).

**Results:**

The enamel microhardness, as a whole, increased (p < 0.05) after a dose of 60 Gy (211.4 KH), mostly in the superficial enamel. There was a significant difference between the values of nonirradiated dentin microhardness (28.9 KH) compared with dentin that was irradiated with doses of 10 Gy (23.8 KH), 20 Gy (25.6 KH), 30 Gy (24.8 KH), and 40 Gy (25.7 KH) (p < 0.05). There was no difference between nonirradiated dentin and dentin irradiated with 60 Gy (p > 0.05). The highest mean value of microhardness (29.9 KH) (p < 0.05) was found in the middle dentin. The groups that were irradiated with doses of 30 and 60 Gy exhibited greater surface changes in their enamel and dentin compared with the nonirradiated groups for all regions, exhibiting an amorphous surface upon increase of the irradiation doses.

**Conclusions:**

The enamel microhardness increased at a dose of 60 Gy, whereas the value of the dentin microhardness did not change. A progressive disruption of enamel and dentin morphology was found with the increased radiation dose.

## Introduction

Head and neck cancers exist at high frequencies in the population, with an incidence of 500,000 new cases per year [1]. In Brazil, the National Cancer Institute (Instituto Nacional do Câncer - INCA) has reported more than 9,000 new cases of childhood cancer per year [2]. Although the incidence of head and neck neoplasms in children is low, the peculiarities of treatment, prognosis, and age-inherent toxicities should be considered [3,4].

Radiation therapy is a therapeutic modality that is widely used to treat head and neck cancer. Although radiation therapy may promote healing, head and neck-irradiated patients are susceptible to oral complications, including mucositis, xerostomia, taste loss, trismus, progressive loss of the periodontal ligament, microvascular alterations, soft tissue necrosis, osteoradionecrosis, and dental caries [5].

Radiation-related caries or “radiation caries” is one of the highest indirect and late effects of radiation in the head and neck region [6]. This complication is a complex and destructive disease that causes severe destruction of the tooth enamel and dentin in head and neck-irradiated patients [5,7,8] and has negative effects on their quality of life [8]. Scientific evidence indicates that patients incur a lifelong risk of developing radiation caries following radiation therapy [7].

The effects of radiation therapy on the onset and progression of a caries lesion might be direct or indirect [8]. The indirect effects of irradiation include changes in the quality and quantity of saliva, difficulty in performing proper oral hygiene, increased intake of cariogenic foods, and changes in the oral microbiota [5,7,9]. Radiation therapy may also exert direct effects on the dental structure, including changes in the crystalline structure, enamel and dentin microhardness, dentinoenamel junction, and acid solubility of the enamel; these effects might be involved in the pathogenesis of the disease [6,10-16].

The direct effects of radiation on the deciduous dentition are still unknown because studies addressing this issue have only been conducted in bovine teeth and in human permanent teeth. Therefore, the aim of the current study was to perform an *in vitro* assessment of the effects of radiation therapy on the mechanical and morphological properties of the enamel and dentin of deciduous teeth using microhardness testing and scanning electron microscopy (SEM).

## Materials and methods

### Sample

Twenty human deciduous molar teeth were used; the teeth were healthy and were either freshly extracted or freshly exfoliated, and they were stored in distilled water at 4°C for periods of less than 1 month. The study was previously approved by the Research Ethics Committee of our school (# 2010.1.1512.58.9).

The teeth were cleaned, polished in a DP-9U2 polishing machine (Panambra/Strues, A/S, Copenhagen, Denmark), refrigerated, and stored in artificial saliva before beginning the experiment. The teeth were mesiodistally sectioned, yielding 2 hemisections, and the vestibular sections were subjected to 2 experiments. In the first experiment, the enamel and dentin microhardness (n = 12 hemisections) was evaluated prior to (control) and after each 10 Gy irradiation, up to a cumulative dose of 60 Gy in the same hemisections. In the second experiment, the enamel and dentin morphologies were evaluated by SEM (n = 8 hemisections), with 2 hemisections being irradiated with a cumulative dose of 30 Gy, 2 hemisections irradiated with a cumulative dose of 60 Gy, and 4 nonirradiated hemisections (control). Radiation therapy was fractioned as follows: 2 Gy *per* day, 5 times *per* week, up to a total dose of 60 Gy, i.e. 30 fractions during 6 weeks, was performed to cover the different ranges used for head and neck radiation therapy.

The dental hemisections were placed in 24-well, acrylic cell-culture plates that were filled with 10 ml of artificial saliva such that all of the specimens received the same direct irradiation per unit area.

### Enamel and dentin microhardness

The initial microhardness of the enamel and dentin was evaluated in the hemisections prior to their irradiation. The test was performed on a microhardness tester (Shimadzu Micro Hardness Tester HMV-2000- Corporation, Kyoto, Japan) with the aid of a diamond indenter for Knoop hardness (KH) by applying a 10-second-long load of 25 gf to the enamel and a 15-second-long load of 10 gf to the dentin.

Indentations were performed in 3 different enamel regions: the first at 50 μm from its outer edge (surface enamel), the second at one-half the thickness of enamel (middle enamel), and the third at 50 μm from the dentinoenamel junction (deep enamel). The dentin indentations were performed at 50 μm from the dentinoenamel junction (surface dentin), at one-half the thickness of dentin (middle dentin), and at 50 μm from the pulp chamber (deep dentin). Three microhardness measurements were conducted by the same calibrated examiner in each selected region, separated by 100 μm in the enamel and 150 μm in the dentin. Indentation for each cumulative, increased irradiation dose was performed close to one another. The average of these measurements was used for the data analysis.

After the initial microhardness evaluation, the dental fragments were irradiated in a Cobalt unit with 1.25 MV photons (Gammatron 580, Siemens, Munich, Germany), a dose rate of 1 Gy/min, and a source-surface distance of 80 cm. We used a dose of 2 Gy/fraction (1 fraction per day, 5 times per week), up to a total dose of 60 Gy (30 fractions over a course of 6 weeks).

Between the cycles of irradiation, the fragments were stored in artificial saliva, which was renewed daily, on an incubator (Olidez CZ, Indústria e Comércio de Aparelhos Hospitalares Ltda., Ribeirão Preto, Brazil) set at 37°C.

The microhardness measurements following irradiation of the enamel and dentin were conducted every 10 Gy of irradiation for up to 30 cycles of irradiation, which is equivalent to a dose of 60 Gy and a period of 30 days. The data exhibited a normal distribution and were analyzed using a two-way analysis of variance (ANOVA) followed by Tukey's test, at a significance level of 5%.

### Enamel and dentin SEM

In total, eight specimens were selected, processed and analyzed by SEM, corresponding to 2 sides irradiated with a cumulative dose of 30 Gy, 2 sides irradiated with a cumulative dose of 60 Gy, and their respective nonirradiated sides (control). The specimens were fixed in a glutaraldehyde solution in cacodylate buffer, cleaned for 10 minutes in an ultrasonic vat (UltrasonicCleaner T-1449-D. Odontobrás Ind. e Com, Ribeirão Preto, Brazil) containing distilled and deionized water, dehydrated in increasing concentrations of ethanol (25%, 50%, 75%, 95%, and 100%), and immersed in hexamethyldisilazane (HMDS) for 10 minutes. Subsequently, the specimens were fixed in stubs with a double-sided adhesive carbon tape (Electron Microscopy Sciences, Washington, USA) and were coated with gold in a vacuum-metallizing machine (SDC 050, Bal-Tec AG, Foehrenwg 16, Balzers, Germany) with a pressure of 0.01 mbar, current of 40 mA, working distance of 50 mm, coating time of 90 seconds, and mean coating thickness of 20 to 30 nm. The specimens were subsequently subjected to analysis by SEM (Microscope Philips XL30 FEG, Eindhoven, Holland).

## Results

### Enamel and dentin microhardness

The lowest mean value of microhardness was found in the surface enamel of the nonirradiated teeth, while the highest mean value was found in the deep enamel of the nonirradiation teeth (p < 0.05). The enamel microhardness increased in surface and middle enamel after irradiation from 180.13 KH to 202.3 KH and 187.82 KH to 217.42 KH, respectively (Table 1). This increase in microhardness was progressive as the irradiation dose augmented from 10 to 60 Gy. In deep enamel, microhardness was not affected by irradiation.

**Table 1:** Mean and standard deviations of the longitudinal microhardness values (Knoop) of the enamel at different depths of the deciduous teeth following irradiation

	**Surface enamel**	**Middle enamel**	**Deep enamel**
**Control (nonirradiated)**	180.13 ± 36.85^bc^	187.82 ± 32.63^B^	206.22 ± 51.41^▲^
**Irradiated 10 Gy**	166.23 ± 27.92^c^	182.89 ± 24.22^C^	205.03 ± 28.39^▲^
**Irradiated 20 Gy**	173.00 ± 42.78^c^	184.09 ± 33.43^C^	196.78 ± 28.70^▲^
**Irradiated 30 Gy**	185.73 ± 25.17^b^	187.11 ± 20.63^B^	205.81 ± 20.95^▲^
**Irradiated 40 Gy**	188.03 ± 43.49^a^	196.70 ± 27.18^AB^	209.72 ± 23.97^▲^
**Irradiated 50 Gy**	197.91 ± 44.04^ab^	205.64 ± 17.65^AB^	208.67 ± 25.62^▲^
**Irradiated 60 Gy**	202.30 ± 17.71^a^	217.42 ± 25.33^A^	214.47 ± 25.99^▲^

Identical symbols (^▲ABC, abc^) denote statistical similarity within the column.

The highest mean values of microhardness were found in the surface and middle dentin of nonirradiated teeth in contrast to the deep dentin, where the lowest mean value of microhardness was found for nonirradiated teeth (p < 0.05).

The microhardness of surface dentin decreased from 30.81 KH found in nonirradiated dentin to 26.54 KH following irradiation up to 40 Gy (p < 0.05). Then, the microhardness increased when teeth were irradiated with 50 and 60 Gy, reaching values similar to those of the nonirradiated teeth (p > 0.05). We found no significant radiation effects on the microhardness of the dentin middle region (p > 0.05), whereas the deep dentin microhardness decreased upon irradiation with 10 Gy (p < 0.05); there was no difference compared with the control after this dose (p > 0.05) (Table 2).

**Table 2:** Mean and standard deviations of the longitudinal microhardness values (Knoop) of the dentin at different depths of the deciduous teeth following irradiation

	**Surface dentin**	**Middle dentin**	**Deep dentin**
**Control (nonirradiated)**	30.81 ± 5.06^a^	31.53 ± 7.94^▲^	24.24 ± 8.47^A^
**Irradiated 10 Gy**	25.22 ± 4.66^b^	27.41 ± 7.52^▲^	18.79 ± 6.18^B^
**Irradiated 20 Gy**	26.07 ± 3.32^b^	31.51 ± 5.59^▲^	19.26 ± 3.33^AB^
**Irradiated 30 Gy**	26.50 ± 5.31^b^	28.86 ± 6.78^▲^	19.03 ± 5.20^AB^
**Irradiated 40 Gy**	26.54 ± 3.53^b^	28.98 ± 5.07^▲^	21.50 ± 8.08^AB^
**Irradiated 50 Gy**	28.48 ± 2.98^ab^	30.82 ± 5.49^▲^	20.86 ± 7.08^AB^
**Irradiated 60 Gy**	27.84 ± 3.13^ab^	30.37 ± 6.76^▲^	21,92 ± 5.78^AB^

Identical symbols (^▲AB, ab^) denote statistical similarity within the column.

### Scanning electron microscopy of the enamel and dentin

**Figure 1:**
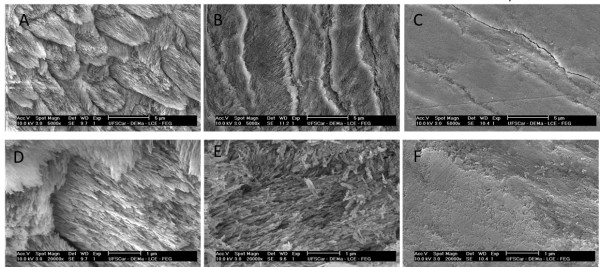
**Electron micrographs of the enamel of the deciduous teeth.** The imagens were obtained by scanning electron microscopy at 5,000x **(A, B, C)** and 20,000x **(D, E, F)** magnifications. **A**, **D**—nonirradiated enamel (control); **B**, **E**—irradiated enamel (30 Gy); **C**, **F**—irradiated enamel (60 Gy).

The enamel of the nonirradiated teeth displayed well-organized prisms, surrounded by interprismatic regions, which were found in cross-sectional and oblique sections. The electron micrographs of the groups that were irradiated with a dose of 30 Gy and 60 Gy revealed a progressive change in the enamel surface in contrast with all of the analyzed regions of nonirradiated enamel. With increasing doses of irradiation, a progressive change was also observed in the prismatic structure of the enamel, impairing the identification of the prisms. Following exposure to 60 Gy, the surface appeared amorphous, precluding the visualization of the prisms and hydroxyapatite crystals, even with the loss of definition of the interprismatic space (Figure 1).

**Figure 2:**
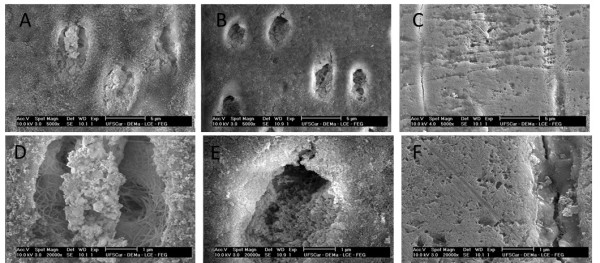
**Electron micrographs of the dentin of the deciduous teeth.** The imagens were obtained by scanning electron microscopy at 5,000x **(A, B, C)** and 20,000x **(D, E, F)** magnifications. **A**, **D**—nonirradiated dentin; **B**, **E**—irradiated dentin (30 Gy); **C**, **F**—irradiated dentin (60 Gy).

Well-defined dentinal tubules with a well-organized collagen network were observed in the nonirradiated teeth (control) by morphological analysis of the dentin. The electron micrographs of the groups that were irradiated with 30 Gy and 60 Gy revealed a progressive change in the surface in contrast with the nonirradiated dentin in all of the assessed regions. Changes in the intertubular and peritubular dentin and degradation of the collagen network occurred upon increasing the doses of irradiation. Upon exposure to 60 Gy, the surface became amorphous, impairing identification of the dentinal tubules, collagen fiber network, and hydroxyapatite crystals (Figure 2).

## Discussion

The results of the present study demonstrate that *in vitro* irradiation of deciduous teeth altered the microhardness and structure of both enamel and dentin. Complications from radiation therapy may vary depending on the general condition of the patient, the tumor characteristics (such as the histological type, location and volume), and radiation features (such as the radiation type, dose, and application rate). The doses for cancer treatment in children range from 50 to 70 Gy, depending on the tumor and the hospital routine protocols [17,18]. However, studies have demonstrated that late effects also depend on the fractionation dose [19,20]. Because treatment protocols have changed considerably over the years and because fractionation doses vary within and between patients, the fractionation dose should also be considered in the evaluation of late effects [19]. The daily dose is normally 2 Gy/day, 5 days/week, interspersed by 2 days without radiation, such that the healthy tissues adjacent to the tumor can recover [5,7]. The maximal dose of 60 Gy, which is used for radiation therapy of head and neck tumors [21], was chosen in the present study to simulate the clinical conditions of radiation therapy.

Additionally, in the current study, the samples were placed in artificial saliva during irradiation to simulate, as precisely as possible, the conditions that are found in the oral cavity [16]. However, other media, including 0.9% saline solution [6,13], distilled water [11,15], or buffered phosphate solution [11], have been used to store teeth in similar studies. Although artificial saliva does not exactly mimic the characteristics of natural saliva, especially in the case of patients undergoing head and neck radiation therapy, who present changes in the flow, secretion, and composition of natural saliva [22], artificial saliva is still considered the most suitable storage medium [16,23].

Studies of the structural changes in enamel and dentin following irradiation are controversial [6,11-13,15,16]. The conflicting results that have been observed are most likely due to the lack of standardization of the methodology in the various studies assessing the direct radiogenic damage to the enamel and dentin. These investigations have used dental substrates of either bovine origin [11-13] or human origin [6,7,15,16], which have been subjected to different doses of radiation [14] and different methods of radiation, mostly with fractional irradiation and some without [12,24].

In the present study, human deciduous teeth were selected because of the increased number of cancer cases in pediatric patients [2], and deciduous and permanent teeth present different morphology, structure, and composition [25]. Of note, compared to permanent teeth, deciduous teeth present a thicker and uniform aprismatic enamel surface [26], thinner enamel and dentin [26,27], higher density of tubules per mm [2] of dentin with diminished intertubular dentin [27]. The novel findings of the present investigation demonstrate that ionizing radiation led to a dose-dependent increase in the enamel microhardness, and a cumulative dose of 60 Gy yielded the highest microhardness values. These findings contrast with previous investigations of permanent teeth that demonstrated either that the microhardness of irradiated enamel is lower than that of nonirradiated enamel [28] or that there is no change in microhardness as a function of radiation [10,11]. However, the present study was conducted using deciduous teeth, and these teeth might respond differently to radiation therapy. Furthermore, ionizing radiation may cause restructuring of the crystal structures of mineralized tissues [10] and thereby modify their physical properties, including the structural microhardness.

In the present study, the enamel microhardness was affected based on the region of the tooth, as the highest values of enamel microhardness were found near the dentinoenamel junction followed by the middle region, with the lowest microhardness values being observed at the surface. Non-dried enamel contains approximately 12% water by volume [12]. In this context, it is noteworthy that this water content is higher in the area of the dentinoenamel junction. Radiation may cause a reduced water content in tissues [29], and tissue dehydration leads to increased organic matrix stiffness and, consequently, to increased microhardness. Specifically, in the dental enamel, this increased stiffness may cause a reduced capacity of the tissue to absorb and dissipate the impact energy due to occlusal loading, making the tissue more friable. In clinical practice, this phenomenon has been observed in patients undergoing head and neck radiation therapy, whose enamel appears to detach from the dentin in regions where these tissues connect, namely, the dentinoenamel junction, which is the region where the greatest increase in enamel microhardness is found.

Tooth enamel is organized into prisms, the orientation of which determines the anisotropic performance of the enamel and affects its mechanical properties [29]. SEM revealed morphological changes in the enamel structure following cumulative irradiation with 30 and 60 Gy, characterized by an increasingly disorganized prismatic structure as the cumulative dose of radiation increased, as previously described for bovine teeth [10]. This change in the enamel crystalline structure has been suggested to be one of the factors related to the increased risk of dental caries following radiation therapy [21].

Although the enamel composition is essentially inorganic, the initial damages from irradiation occur in the organic portion of the enamel, that is, in the interprismatic space, via the oxidation of water molecules into hydrogen peroxide and hydrogen free radicals that denature the organic components [24]. Consequently, the mechanical properties and integrity of the enamel are affected [6]. However, we demonstrated in the present study that irradiation also caused changes in the prismatic structure of the enamel, suggesting that the clinically observed radiation effects result from changes in both organic and inorganic compounds in the enamel.

The clinical extrapolation of findings from the *in vitro* or *in situ* studies that have evaluated the structure, demineralization, and dissolution of irradiated tooth enamel should be cautiously performed, given the difficulties of mimicking the *in vivo* and *in situ* clinical alterations and the individual responses of each patient to the effects of radiation therapy [6,7,11,15,16,30]. Notwithstanding, in the present study, the measured changes in the physical and mechanical properties of the tissue indicate that disruption of the enamel and its superficial microhardness contributes to the clinically observed alterations.

The individual analysis of each region of dentin revealed that irradiation only affected the longitudinal microhardness in the region near the dentinoenamel junction, which was lower than the microhardness of the same region in nonirradiated teeth. This reduction confirms the results from previous studies, which used radiation doses of up to 70 Gy, albeit with different irradiation protocols [9,12].

The decrease in the irradiated dentin microhardness can be explained because the changes in organic components within the dentinal tubules were considered the main reasons for the reduced physical strength of the dentin following the *in vivo* or *in vitro* irradiation of permanent teeth [6]. Dentin contains 12% water content by volume, enabling an increased production of free radicals and hydrogen peroxide as a function of radiation [27]. These compounds denature the organic content of dentin, decreasing the internal stability of this tissue [12].

By SEM analysis, morphological changes were found in the dentin of the deciduous teeth following cumulative irradiation with 30 and 60 Gy. There was increased disruption, characterized by degradation of the collagen network and changes in the intertubular and intratubular dentin with the increased cumulative dose of radiation, in contrast with the nonirradiated dentin. The obliteration of dentinal tubules in irradiated permanent teeth has been attributed to the degeneration of odontoblastic processes, which are due to direct radiogenic damage to the tissue caused by the action of free radicals [14]. The changes in the dentin structure, specifically, the loss of orientation of the dentinal tubules, lead to reduced microtensile strength [15].

To the best of our knowledge, this is the first report of the effects of radiation therapy on the morphology and mechanical properties of deciduous teeth. Based on the findings of the current study, we suggest that the increased risk of radiation caries in the deciduous teeth of children undergoing head and neck radiation therapy are due not only to the well-known salivary, dietary, and microbiological changes but also to changes in the dental morphology and enamel microhardness. This study is an *in vitro* investigation and therefore further studies that assess other variables and include other levels of research (such as clinical trials) are necessary to confirm that irradiation leads to structural changes in the dental substrate and might thereby cause many dental problems in pediatric cancer patients.

## Conclusions

The irradiation of deciduous teeth affected the longitudinal microhardness of the enamel and dentin as a function of the dose and the irradiated region. In the enamel as a whole, the microhardness increased following a cumulative dose of 60 Gy, regardless of the region analyzed. In the dentin, there was no change in the microhardness values following irradiation, regardless of the region analyzed. A morphological disruption occurred in the enamel and dentin, which began to exhibit an amorphous surface that complicated identification of the enamel prisms and the dentinal tubules, respectively.

## Competing interests

The authors declare that they have no competing interests.

## Authors’ contributions

TSM, AMQ, RGPD, and HFO participated in study design. FWGPS and PNF contributed to conception and organization of the study. TSM conducted the experiments. TSM, AMQ, PNF, FWGPS and RGPD manuscript and conducted data analysis. LABS, RABS, HFO and FWGPS participated in interpretation of the results. All authors have read, reviewed, and approved the final manuscript.
